# The effects of the NMDAR co-agonist d-serine on the structure and function of optic tectal neurons in the developing visual system

**DOI:** 10.1038/s41598-023-39951-4

**Published:** 2023-08-17

**Authors:** Zahraa Chorghay, Vanessa J. Li, Anne Schohl, Arna Ghosh, Edward S. Ruthazer

**Affiliations:** 1 Montreal Neurological Institute-Hospital and Department of Neurology and Neurosurgery, McGill University, 3801 Rue University, Montréal, QC H3A 2B4 Canada; 2 MILA, 6666 Rue St Urbain, Montréal, QC H2S 3H1 Canada

**Keywords:** 3-D reconstruction, Fluorescence imaging, Time-lapse imaging, Development of the nervous system, Neural circuits, Synaptic transmission

## Abstract

The N-methyl-d-aspartate type glutamate receptor (NMDAR) is a molecular coincidence detector which converts correlated patterns of neuronal activity into cues for the structural and functional refinement of developing circuits in the brain. d-serine is an endogenous co-agonist of the NMDAR. We investigated the effects of potent enhancement of NMDAR-mediated currents by chronic administration of saturating levels of d-serine on the developing *Xenopus* retinotectal circuit. Chronic exposure to the NMDAR co-agonist d-serine resulted in structural and functional changes in the optic tectum. In immature tectal neurons, d-serine administration led to more compact and less dynamic tectal dendritic arbors, and increased synapse density. Calcium imaging to examine retinotopy of tectal neurons revealed that animals raised in d-serine had more compact visual receptive fields. These findings provide insight into how the availability of endogenous NMDAR co-agonists like d-serine at glutamatergic synapses can regulate the refinement of circuits in the developing brain.

## Introduction

During the development of functional circuits, neuronal processes elaborate and establish coarse topographic maps, then undergo synaptic and structural refinement to enable precise connectivity^1^. The N-methyl-d-aspartate type glutamate receptor (NMDAR) appears to play an evolutionarily conserved role in the activity-dependent selection of inputs for refinement^2^. While NMDARs are heterogenous in their composition, they classically require simultaneous ligand binding of glutamate and a co-agonist, either glycine or d-serine^3^, alongside sufficient depolarization to relieve the magnesium block of the ion channel pore^4,5^. The concurrent ligand-binding and membrane depolarization requirements for channel conductance make NMDARs ideal for detection of the temporal correlation of convergent inputs^6^.

This suggests a model whereby NMDAR activation can convert patterned neuronal activity into signaling cascades that direct the refinement of topographic maps. Correlated activity has been shown to mediate synaptic strengthening and promote axon arbor stabilization, prolonging branch lifetimes and suppressing branch dynamics^7–9^. Conversely, uncorrelated activity promotes axonal branch destabilization, including increased branch addition, loss, and elongation^10^. In a number of models, loss of NMDAR function perturbs arbor growth and dynamics of both axons and dendrites, leading to disorganization of afferent projections during the development of topographic maps^11–22^.

d-Serine is found endogenously in the brain in a similar distribution to that of NMDARs^23,24^ and enhances NMDAR-dependent synaptic transmission^25–27^. d-serine has been implicated in hippocampal long-term potentiation^27–30^ and depression^31–33^, as well as aspects of learning and memory^34,35^. In the nervous system, whether glia or neurons are the primary source of d-serine release remains controversial^36–38^, and likely depends on the brain region, developmental stage, and presence of pathology^39,40^.

The role of NMDARs in developmental plasticity has largely been characterized through loss-of-function manipulations^1,7–22,41,42^, which may lack specificity if they disrupt normal network activity by reducing overall neuronal excitation. In contrast, administration of d-serine offers a pharmacological manipulation that enhances existing NMDAR currents while preserving the requirement for glutamate release^43^. Therefore, we used d-serine administration as a gain-of-function manipulation to study the effects of NMDAR-specific signal enhancement on circuit development.

Under physiological conditions, NMDAR function is modulated by the availability of co-agonist^43,44^. Pharmacological blockade of the co-agonist binding site results in a full loss of NMDAR conductance, limiting the value of such experiments for understanding the contributions of endogenous d-serine to circuit development. Chronic exposure to saturating amounts of d-serine bypasses the endogenous regulation of co-agonist availability. We previously demonstrated that exogenous d-serine administration promotes the functional maturation of glutamatergic synapses through α-amino-3-hydroxy-5-methyl-4-isoxazolepropionic acid type glutamate receptor (AMPAR) trafficking, and stabilizes axonal arbor structure in the Xenopus tadpole visual system^43^. However, this study did not address the effects of d-serine rearing in development on postsynaptic dendritic remodeling, synaptogenesis and the fine-tuning of visual responses. Here, we use chronic, saturating d-serine administration to examine structure and function of postsynaptic neurons in the optic tectum. We found that d-serine administration led to more compact and stable dendritic arbor morphology specifically in immature tectal neurons, increased synapse density, and resulted in sharper visual receptive fields in the optic tectum.

## Materials and methods

### Husbandry and animals

The study was approved by the Animal Care Committee of the Montreal Neurological Institute at McGill University. All procedures were carried out in accordance with Canadian Council on Animal Care guidelines.

For generation of tadpoles, female adult albino *Xenopus laevis* frogs (RRID:XEP_Xla300) from our in-house breeding colony were primed with 50 IU pregnant mare serum gonadotropin (PMSG; Prospec Bio HOR-272). After 3 days, these female frogs were injected with 400 IU human chorionic gonadotropin (hCG; Sigma-Aldrich CG10; RRID:SCR_018232).

#### Natural fertilization (Figs. 1, 2, 3)

Males were injected with 150 IU hCG on the same day as females and placed together to induce amplexus. Eggs were collected the following day.

#### In vitro fertilization with mRNA injection (Fig. 4)

Eggs from primed females were collected for in vitro fertilization with sperm from male albino frogs. Microinjection of GCaMP6s and mCherry messenger ribonucleic acid (mRNA) into one blastomere of two-cell stage embryos was performed as previously described^1,21^. Briefly, a mixture of purified GCaMP6s (500 pg) and mCherry (250 pg) mRNA in 2 nL RNase-free water was pressure injected into one blastomere of two-cell stage embryos using a calibrated glass micropipette attached to a PLI-100 picoinjector (Harvard Apparatus). Several days after injection, we screened for animals with hemilaterally restricted mCherry expression and high levels of GCaMP6s fluorescence for use in calcium imaging experiments. Since retinal ganglion cell (RGC) axons project contralaterally to the optic tectum, GCaMP6s labels RGC axon terminals and postsynaptic tectal cells in opposite hemispheres of these animals, allowing us to perform calcium imaging of either the RGCs or the tectal cells in each tectal lobe.

#### Tadpole rearing

For all experiments, tadpoles were reared in glass bowls kept in biological oxygen demand incubators set to 21 °C with a 12 h:12 h light:dark cycle. Rearing medium was 0.1X Modified Barth’s Solution with HEPES (MBSH; 88 mM NaCl, 1 mM KCl, 2.4 mM NaHCO_3_, 0.82 mM MgSO_4_ × 7H_2_O, 0.33 mM Ca(NO_3_)_2_ × 4H_2_O, 0.41 mM CaCl_2_, 10 mM HEPES, pH 7.4).

### Constructs

pCAG-Cre, pCALNL-EGFP, pCALNL-DsRed are a generous gift from CL Cepko and are currently available through Addgene (plasmids 13775, 13770, 13769). All plasmids were grown in DH5a competent cells (Life Technologies) and purified using endotoxin-free maxiprep kits (Qiagen)^45^.

### mRNA synthesis

To synthesize the mRNA for blastomere injections, GCaMP6s (Addgene plasmid 40753) and mCherry (plasmid gift of K. Murai) were each cloned into the pCS2 + vector. The GCaMP6s plasmid was cut with NotI/Klenow fill in/BglII, the mCherry plasmid was cut with BamHI/EcoRV, and the pCS2 + vector was cut with BamH1/SnaB1. For mRNA synthesis, the plasmids were linearized with NotI, and capped mRNA of GCaMP6s and mCherry were transcribed with the SP6 mMessage mMachine Kit (Ambion, Thermo Fisher).

### Electroporation (Figs. 1, 2, 3)

**Figure 1:**
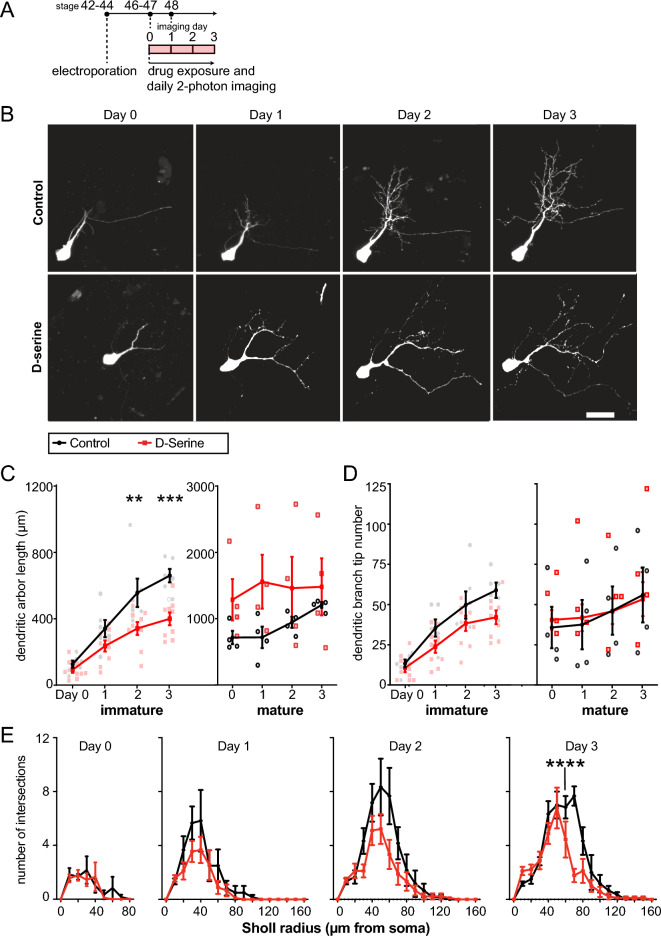
Growth of dendritic arbors of tectal cells in animals reared in d-serine over 4 days. (**A**) Experimental design. Tadpoles at stage 42–44 were electroporated, screened for single-cell GFP expression 48 h later at stage 46–47, and the GFP + cell was imaged daily. After a baseline image on day 0, the animals were reared in control or d-serine medium, and images collected every day for 3 more days. (**B**) Z-projections of representative tectal neurons. Scale bar: 20 µm. (**C**, **D**) Quantification of the (**C**) length and (**D**) number of tips of the dendritic arbors from tadpoles reared in d-serine (red squares) compared to control (black circles), subdivided into immature (total dendritic arbor length < 500 µm on day 0) or mature (≥ 500 µm on day 0). Immature cells were labeled by CREMSCLE and mature cells by single-cell electroporation. For immature cells, total dendritic arbor length and branchtip number were reduced in d-serine compared to control. [RM ANOVA interaction *p < 0.05, Šídák's multiple comparisons post-hoc test Day 2 **p < 0.005, Day 3 *p < 0.05. 1 cell per animal, immature cells: n = 6 cells for d-serine, n = 9 for control, mature cells: n = 4 cells for both groups] (**E**) Sholl analysis of CREMSCLE cells shows tectal dendritic arborization occurs closer to the soma in animals reared in d-serine. [multiplicity-corrected RM ANOVA interaction, Day 3 p < 0.0005, Šídák's post-hoc test for multiple comparisons ****p < 0.0001. 1 cell per animal analyzed with n = 9 cells for d-serine, n = 6 for control].

Tadpoles at stage 42–44 were anesthetized in MS222 (0.02% in 0.1% MBSH) and placed on a Kimwipe under a dissecting microscope. Cre-mediated single-cell labelling by electroporation (CREMSCLE) for high-efficiency, sparse labelling of the optic tectum was performed^41^. For daily imaging (Fig. 1), Cre-recombinase and Cre-dependent EGFP at a ratio of 1:4000 (2.5 × 10^–4^ µg/µL pCAG-Cre, 1 µg/µL pCALNL-EGFP) were injected intraventricularly together with Fast Green dye for visualization. To count synapses per cell (Fig. 3C,D), we co-electroporated Cre-recombinase and Cre-dependent dsRed at a ratio of 1:4000 (2.5 × 10^–4^ µg/µL pCAG-Cre, 1 µg/µL pCALNL-dsRed) and pPSD95-GFP (1 µg/µL). Immediately after the intraventricular injections of the plasmids, a pair of custom-made platinum plate electrodes, connected to an electrical stimulator (SD 9, Grass Instruments), was placed on each side of the brain to deliver current pulses: 38 V, 1.6–3 ms, 2 pulses at reverse polarity 1 s apart. A 3 µF capacitor was connected in parallel to generate an exponential decay current pulse.

While CREMSCLE was used to target smaller, more immature neurons, we used juxtacellular single-cell electroporation^46^ to study the morphologies of a broader range of tectal cells. A borosilicate glass micropipette (Sutter Instruments) containing the plasmid (1 µg/µL EGFP) was gently introduced into the brain of anesthetized stage 44–45 tadpoles. A 50 V train of 1 ms pulses at 200 Hz was applied for 0.5 s through the micropipette, with pulse trains repeated twice to increase delivery of the plasmid.

We used the experimental protocol for d-serine administration published previously^43^. 48 h after electroporation (stage 46–47), animals were screened for brightly labelled, well-separated tectal cells, then returned to be reared in an isolated well that contained control MBSH, or MBSH supplemented with 100 µM d-serine (Tocris), or MBSH with 100 µM d-serine and 10 µM MK-801. Animals reared in these media for up to 3 d were imaged as specified below.

### In vivo two-photon imaging for morphology (Figs. 1, 2, 3)

Excitation light at 910 nm (EGFP, GCaMP6s) or 990 nm (dsRed) was produced by a Mai Tai BB Ti:Sapphire or an InSightX3 femtosecond pulsed IR laser (Spectra Physics).

#### Daily imaging (Fig. 1)

Animals were screened for bright, sparse EGFP expression in the optic tectum, anesthetized in MS222 (0.02% in 0.1X MBSH), placed in a custom-made Sylgard chamber that fit the tadpole’s body, and secured under a cover glass. Z-series images of single tectal neurons were acquired daily on an Olympus FV300 microscope modified for multiphoton imaging with an Olympus LUMPLAFLN 60X water-immersion objective (1.0 NA). Z-series optical sections were collected at 1 µm intervals using Fluoview software (version 5.0). After imaging, the animals were individually placed in wells of a 6-well plate in d-serine or control rearing medium. Images were collected every day for 4 days, with the animals returned to their respective well and the rearing media changed daily. All image z-stacks were denoised using CANDLE non-local means denoising software implemented in MATLAB (MathWorks)^47^, and three-dimensional reconstructions of single neurons were performed using Imaris 6.0 (Bitplane). Some neurons from the control group appeared previously in a methods paper^45^.

#### Short interval imaging (Fig. 2)

Animals selected for bright, sparse EGFP expression in the optic tectum were placed in isolated wells containing d-serine or control rearing medium. 24 h later, the rearing medium was refreshed, and 48 h after screening (corresponding to day 2 of daily imaging, stage 48), animals were imaged. Tadpoles were immersed in 2 mM pancuronium bromide (Tocris) for 3–5 min, embedded in 0.8% w/v UltraPure low melting point agarose on a petri dish, and the petri dish filled with rearing medium.

Dendritic arbors were imaged with an Olympus XLUMPlanFLN 20X water-immersion objective (1.0 NA) mounted on a commercial high-speed resonance scanner-based multiphoton microscope (Thorlabs) with piezoelectric objective focusing (Physik Instrumente). Z-series optical sections were collected at 1 µm intervals using ThorImage software (version 3.0 +). After an initial image was taken (timepoint 0 min) images were collected every 10 min for 1 h while the animal was presented with 10 ms full-field light flashed from a red luxeon LED at 1 Hz. This stroboscopic visual stimulation was controlled by an Arduino Uno R3 board and a custom Matlab script. All image z-stacks were denoised with CANDLE, the four-dimensional manual reconstruction for dynamic morphometric analysis performed using Dynamo software implemented in MATLAB^48^, and analyzed using a custom Python script https://github.com/fieryzarzar/morphology_timecourse_analysis.

### Immunohistochemistry for anatomical synapses (Fig. 3A–C)

Following a previously published procedure^49^, animals raised in d-serine or control rearing medium for 48 h (corresponding to Day 2 of daily imaging, stage 48) were anesthetized in 0.02% MS-222, fixed by immersion in 4% paraformaldehyde (Cedarlane/EMS 15735-30-S) in phosphate buffered saline (PBS) for 1 h at room temperature, transferred to ice-cold 100% methanol, and post-fixed overnight at – 20 °C. Samples were then washed for 1 h in a solution of 100 mM Tris/HCl, pH 7.4 with 100 mM NaCl. For infiltration and cryoprotection, the samples were incubated overnight at room temperature in a solution of 15% fish gelatin (Norland HP-03) with 15% sucrose, and subsequently in 25% fish gelatin with 15% sucrose. Samples were embedded and frozen in a solution of 20% fish gelatin with 15% sucrose for cryosectioning. Horizontal sections were collected at 20 µm thickness on a cryostat and directly mounted onto Superfrost-plus slides (Fisher).

Sections were processed for antigen retrieval with 1% sodium dodecyl sulfate (SDS) in PBS for 3 min at room temperature. Slides were incubated with blocking solution (10% bovine serum albumin and 5% normal goat serum in PBS) followed by rabbit anti-GluA1 (1:200; Abcam ab109450 RRID:AB_10860361) and mouse anti-SV2 (1:1000; Developmental Studies Hybridoma Bank sv2-2a RRID:AB_2315387), and labelled with secondary antibodies Alexa-555 goat anti-rabbit IgG (1:200; Invitrogen A-21428 RRID:AB_141784) and Alexa-647 goat anti-mouse IgG (1:200; Invitrogen A21236 RRID:AB_2535805). Confocal images of the optic tectum were acquired with a 20x/0.75 CS2 objective on a Leica SP8 confocal microscope.

#### Synapse quantification (Fig. 3C)

For synapse quantification, a previously published approach^49^ was used. Briefly, analysis was performed on the optical sections 3 μm beneath the cut surface of the histological section in fields devoid of excess vasculature or sectioning irregularities. 20 μm × 20 μm fields were selected from the neuropil region from each imaged tectal hemisphere. Images were pre-processed using FIJI with background subtraction (10 px radius rolling ball) then median filtering (2 px radius), followed by Moments auto-thresholding for each channel. To identify synapses, the logical AND of the SV2 (presynaptic) and GluA1 (postsynaptic) channels was taken, then the Analyze Particles function with the size criterion of 0.1–5.0 μm^2^ area was applied to estimate numbers of puncta with pre- and postsynaptic labelling. Analysis was performed blind to experimental condition.

#### Synapse quantification by live imaging (Fig. 3D–F)

Animals screened for bright, sparse dsRed expression were raised in either d-serine or d-serine plus MK-801 or control rearing medium for 48 h (corresponding to Day 2 of daily imaging, imaged at stage 48). Live animals were imaged with the same setup as for daily imaging. Z-series optical sections were collected at 1 µm intervals using Fluoview software (version 5.0) for both the red (dsRed) and green (PSD95-GFP) channels. All image z-stacks were processed with FIJI software using the background subtraction (rolling ball 10 µm) and median (radius 2 µm) filtering functions. Images were made binary using MaxEntropy and Moments auto-thresholding for the dsRed and PSD95-GFP channels, respectively. To identify synapses, the Analyze Particles function with a size criterion of 0.1–5.0 μm^2^ was applied to identify synaptic puncta marked with PSD95-GFP. The logical AND of the two channels using the dsRed channel as a dendritic mask, identified synaptic puncta within the defined dendritic volume. Cell somata and axons were excluded from analysis. Analysis was performed blind to experimental condition.

### In vivo two-photon calcium imaging for retinotopic mapping (Fig. 4)

Using a previously published procedure^1^, animals selected for bright GCaMP6s and mCherry expression restricted to one lateral half of their body were transferred to d-serine or control rearing medium from stage 37 onwards and imaged a week later at stage 48, since retinal ganglion cell (RGC) axons arrive in the tectum after stage 37^50^.

Tadpoles were immobilized by immersion in 2 mM pancuronium bromide and immobilized in 1% low-melting point agarose in a custom chamber with a glass coverslip window on one side, through which the animal could view visual stimuli presented on an LCD screen. The LCD display area measured 6.5 cm (width) × 4 cm (height). The tadpole was positioned so the eye was 2.2 cm from the screen, aligned to the center of the bottom edge of the display area. From this viewpoint, the display area spanned roughly 110° visual angle in azimuth and 80° in elevation.

Calcium fluorescence images were captured with a high-speed resonance scanner-based two-photon microscope (Thorlabs) with piezoelectric focusing (Physik Instrumente) of a 1.0-numerical aperture 20 × water immersion Nikon objective. An excitation wavelength of 910 nm was used for GCaMP6s, and emission signal was collected through a 525/50 bandpass filter. A #29 Wratten filter (Kodak) was installed on the LCD screen to prevent light from the display from interfering with the calcium signal. Custom MATLAB scripts based on the Psychophysics Toolbox (RRID: SCR_002881)^51–53^ were used to generate the visual stimuli and synchronize stimulus presentation with image capture. Visual stimuli were presented monocularly, and calcium signal was imaged from the tectum contralateral to the stimulated eye. Images (512 × 512 pixels, 0.496 µm per pixel) were collected from a single optical section at 15 Hz or from three to four optical sections (with one to two flyback frames) at 6 Hz.

Processing and analyses of calcium imaging data were performed with custom scripts in MATLAB (RRID: SCR_001622) and Fiji (RRID: SCR_002285). For analyses comparing two separate recordings, images were aligned using the MATLAB *imregtform()* library function or the NoRMCorre algorithm for nonrigid motion correction^54^.

Visual field representation in the tectum was then estimated with “grid mapping”^1^. Briefly, the animal was presented with vertical or horizontal 18°-wide dark bars at 5 equidistantly spaced positions along the azimuth or elevation axis respectively, in a randomized fashion. For each pixel, an “optimal stimulus position” was calculated based on the pixel’s weighted average ΔF/F_o_ response to each stimulus position to estimate the pixel’s receptive field center. Cell body ROIs were automatically segmented using Cellpose^55^ and ∆F/F_o_ responses were averaged within each ROI. “Receptive field sharpness” was quantified as the average ∆F/F_o_ response to the two stimulus positions closest to the “optimal stimulus position” divided by the average response to the remaining stimulus positions in the periphery. Only cell bodies with maximal stimulus response ∆F/F_o_ > 2 and optimal stimulus positions falling between the three central stimulus positions were evaluated. Cell bodies smaller than 30 pixels and animals with fewer than 30 cell bodies fitting the evaluation criteria were excluded.

### Statistical analysis

Analyses were conducted in accordance with ARRIVE guidelines. Statistical analyses were performed in GraphPad Prism 8.0. Normality of the data distributions was confirmed using the Shapiro–Wilk test. The details for the statistical tests for each experiment can be found in the figure legends. All quantification is graphed as mean ± SEM, unless indicated (Supplementary Information).

### Custom code

The custom code is available on Github for the dynamics of dendritic filopodia https://github.com/fieryzarzar/morphology_timecourse_analysis and for retinotopic mapping https://github.com/RuthazerLab/XenMap.

## Results

### Tectal dendrite elaboration of immature neurons is reduced by d-serine rearing

To visualize dendritic arbor morphology of neurons in the optic tectum, we electroporated tectal cells to sparsely express EGFP, then performed daily in vivo two-photon imaging over four days. After collecting baseline two-photon laser scanning microscope images of individually labeled tectal neurons (Day 0, stage 46–47), animals were reared in 100 μM d-serine, which enhances retinotectal NMDAR currents^43^. Over the following 3 day (stage 48), cells were imaged every 24 h (Fig. 1A,B). For neurons with initially less mature dendritic arbors (< 500 μm initial arbor length), but not those with larger arbors (≥ 500 μm), we observed a significant reduction in dendritic arbor length in d-serine reared animals compared to controls, apparent by by 2 days (Fig. 1C), but no significant differences in the numbers of dendritic branch tips (Fig. 1D). We therefore focused our morphological analysis on immature neurons.

CREMSCLE is a bulk electroporation method for sparse labelling based on co-electroporation of low-concentraton cre recombinase and floxed-stop EGFP plasmids^45^. Because plasmid is injected into the brain ventricle, it preferentially labels recently differentiated, immature cells close to the periventricular proliferative zone of the tectum. Sholl analysis of these initially immature neurons revealed that d-serine rearing for 3 days reduced the elaboration of the dendritic arbor far from the cell body compared to cells in control animals (Fig. 1E). Overall, immature neurons exposed to d-serine developed to be more compact than controls.

### Tectal dendritic arbors are structurally stabilized by D-serine-rearing

**Figure 2:**
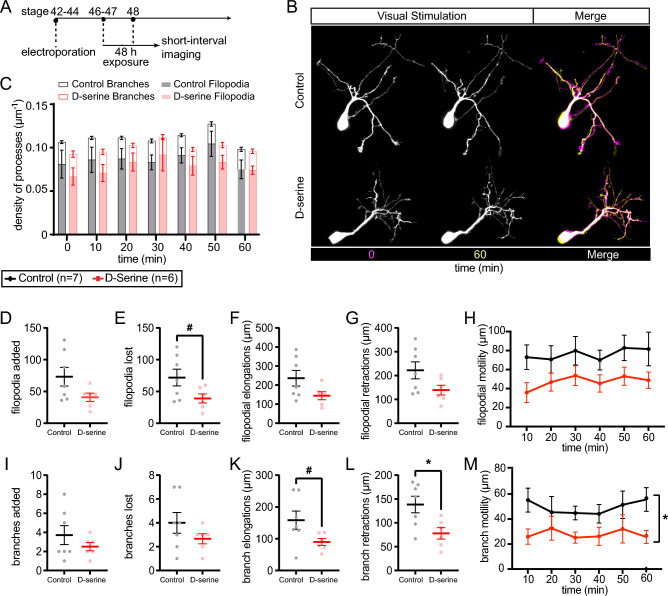
Filopodia and branch dynamics of tectal cell dendritic arbors in animals reared in d-serine for 48 h. (**A**) Experimental design. Tadpoles at stage 42–44 were electroporated, screened for GFP expression 48 h later at stage 46–47, and reared in control or d-serine medium for 48 h to stage 48. After the initial image, cells were imaged every 10 min for 1 h as tadpoles were visually stimulated with 1 Hz strobe flashes. (**B**) Representative images of dendritic arbors during imaging and a final merged image overlaying timepoints 0 (magenta) and 60 (yellow) min. Scale bar: 10 µm. (**C**) Density of processes counted per timepoint for filopodia (dendritic processes < 10 µm length; shaded) and branches (≥ 10 µm length; unshaded) from control (black) and d-serine (red) arbors. Density was calculated as the number of dendritic processes divided by arbor length. (**D**) Number of filopodia added to the dendritic arbor over 1 h of imaging. (**E**) Number of filopodia lost showed a trend towards a decrease in d-serine arbors compared to control. [t-test ^#^p = 0.06] (**F**) Length of filopodial elongations and (**G**) retractions over 1 h of imaging. (**H**) Filopodial motility (sum of elongations and retractions) per timepoint. (**I**–**M**) Quantification for branches, including (**I**) number added and (**J**) lost over 1 h. (**K**) Branches showed a trend towards elongating less over 1 h in d-serine arbors compared to control. [t-test ^#^p = 0.06] (**L**) Length of branch retractions over 1 h was significantly less in the d-serine group [t-test *p < 0.05], as was the (**M**) branch motility per timepoint [RM ANOVA main effect of treatment *p < 0.05]. 1 cell per animal analyzed with n = 6 for d-serine, n = 7 for control.

We hypothesized that these morphological differences could have resulted from premature stabilization of the dendritic arbors of immature neurons in d-serine. To test this idea, branch dynamics were investigated with short-interval imaging every 10 min for 1 h in animals exposed to visual stimulation in the form of 1 Hz stroboscopic flashes (“strobe”). As daily imaging revealed a significant impact on arbor length by 48 h of d-serine exposure (Fig. 1C), we next imaged dynamic remodeling of dendritic arbors in stage 48 tadpoles that had been reared in d-serine for 48 h (Fig. 2A,B). Dendritic processes were categorized as filopodia (< 10 µm length) or branches (> 10 µm) and analyzed separately since these dendritic compartments have been shown to have distinct cytoskeletal elements, activity-dependent growth, and links to synaptogenesis^48,56^.

d-serine rearing did not appear to change the overall density of dendritic processes (number of processes divided by arbor length), regardless of whether the processes were filopodia or branches (Fig. 2C). The total numbers of added and lost filopodia (Fig. 2D,E) and branches (Fig. 2I,J) were not significantly different between d-serine reared and control arbors, although a trend toward fewer dynamic events in d-serine-reared animals was apparent. We also quantified filopodial and branch elongations (Fig. 2F,K) and retractions (Fig. 2G,L) over the hour, as well as their total motility per timepoint (Fig. 2H,M). Quantification of filopodial growth showed no significant differences between the two groups (Fig. 2F,G). However, dendritic branches in d-serine-reared animals exhibited a trend towards elongating less (Fig. 2K) and showed significantly less retraction (Fig. 2L) over the hour of imaging. Overall, d-serine exposure significantly reduced branch motility per timepoint over the hour of imaging (Fig. 2M). These observations of arbor dynamics suggest that dendritic branches chronically exposed to d-serine have a decreased tendency to elongate and retract during visual stimulation, leading to dendritic arbors that are more stable over time. Notably, the effects of d-serine on stabilization are observed in more mature processes (branches) which are more likely to bear synapses. These observations support the hypothesis that NMDAR signaling enhancement leads to hyperstability of the dendritic arbor.

### Retinotectal synaptic density is increased with d-serine exposure

**Figure 3:**
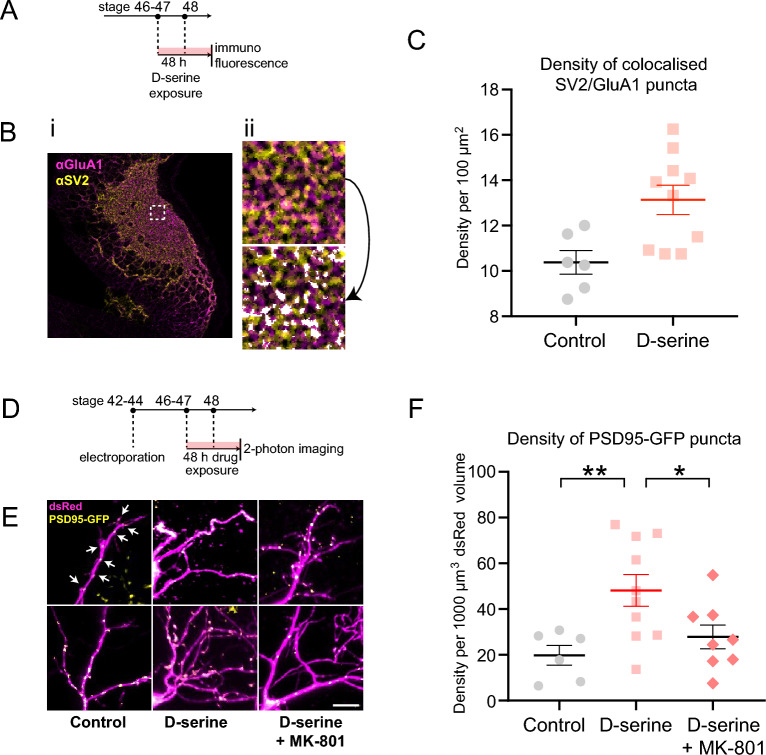
Synaptic density in the tectum of animals reared in d-serine for 48 h. (**A**) Experimental design: Tadpoles at stage 46–47 were reared in control or d-serine medium for 48 h to stage 48, then fixed, sectioned and immunostained for SV2 and GluA1 for confocal imaging. (**B**) Colocalized puncta of SV2 (presynaptic) and GluA1 (postsynaptic) immunofluorescence on brain sections. For a sample field, (i) the tectal neuropil is shown, (ii) zoomed into a dashed 20 µm × 20 µm region-of-interest (top) with automated processing to identify “anatomical synapses” (bottom, white overlay, see “Materials and methods”). (**C**) Anatomical synapse density in the optic tectum was significantly elevated for animals reared in d-serine. [t-test *p < 0.05. 2–3 fields per tectal hemisphere were analysed i.e. at least 5 fields per animal from n = 5 d-serine, 3 control animals]. Only colocalized puncta that fit the synapse size criterion of 0.1–5.0 µm^2^ were included. (**D**) Experimental design: Tadpoles at stage 42–44 were electroporated, screened for dsRed and PSD95-GFP expression 48 h later at stage 46–47, reared in control, d-serine, or d-serine + MK-801 medium for 48 h and imaged at stage 48. (**E**) Magnified dendritic arbors of tectal neurons expressing PSD95-GFP + puncta (yellow) in dsRed + cells (magenta). Scale bar: 10 µm. Arrows: examples of synaptic puncta. (**F**) Quantification of the densities of PSD-95 puncta per volume of dendritic arbor for single tectal neurons reveals that rearing in d-serine increases synapse density, which is prevented when NMDARs are blocked by MK-801. [one-way ANOVA with Dunnett’s post-hoc test for multiple comparisons **p < 0.01, *p < 0.05. n = 6 control, 10 d-serine, 8 d-serine + MK-801 cells].

Since dendritic branch morphogenesis was affected by d-serine-rearing, we next investigated whether synaptogenesis may also be affected. We examined the effects of chronic d-serine on synapse density using immunohistochemical labelling of presynaptic (SV2) and postsynaptic (GluA1) markers on cryostat sections of optic tectum from animals reared in d-serine for 48 h (from stage 46–47 to stage 48). Quantification of “anatomical synapses’’ was based on the overlapping juxtaposition of these pre- and postsynaptic markers in confocal images of tectal neuropil^49^. We found that anatomical synapse density was significantly increased in the tectal neuropil following 2 day of d-serine exposure (Fig. 3A–C).

Taken together, our observations of more compact, stable dendritic arbors and increased synapse number in the tectal neuropil of d-serine-reared animals suggest that their individual tectal dendrites should have increased density of synaptic inputs. We therefore electroporated tectal cells at stage 42–44 to express the synaptic marker postsynaptic density protein 95 fused to EGFP (PSD95-GFP) along with sparse single-cell expression of dsRed by CREMSCLE. Animals with suitable labeling were then reared for 48 h in medium containing d-serine (Fig. 3D). Quantification of PSD95-GFP punctum density in dsRed-labelled neurons confirmed that d-serine-reared cells had significantly greater numbers of synaptic puncta per dendritic volume (Fig. 3E,F). To exclude the possibility that d-serine may be acting via non-NMDA receptor targets, such as synaptogenic glutamate *delta* receptors^57,58^, some animals were reared in d-serine plus the NMDAR channel blocker MK-801 (10 µM). Addition of MK-801 prevented the increase in synapse density, indicating that the observed effect of d-serine is NMDAR-dependent.

### Retinotectal map refinement is altered by d-serine-rearing

**Figure 4:**
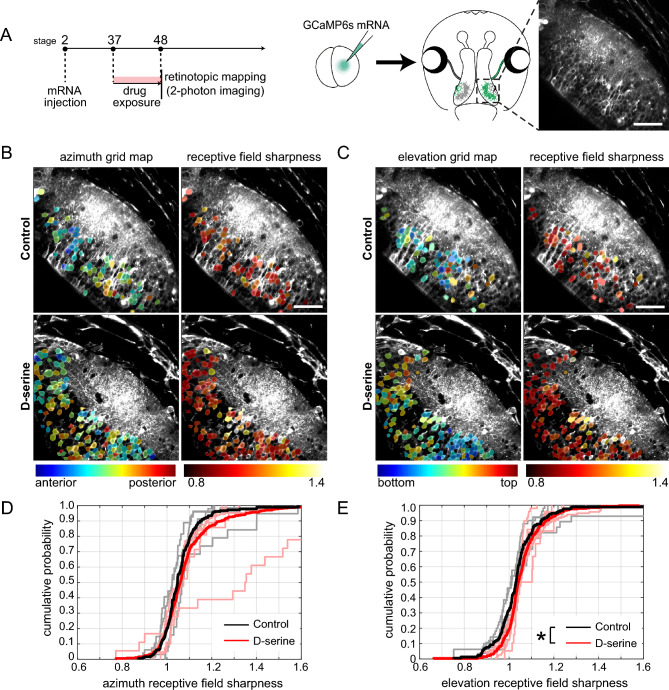
Postsynaptic retinotopic maps in stage 48 animals reared in d-serine from stage 37. (**A**) Experimental design. mRNA for GCaMP6s was injected into one blastomere at the two-cell stage to generate tadpoles with mosaic GCaMP6s expression restricted to half the animal. Tadpoles were screened at stage 37 for GCaMP6s expression, then reared in control or d-serine for two-photon imaging of calcium fluorescence in the optic tectum at stage 48. Scale bar: 50 µm. (**B**, **C**) Representative images showing receptive field center locations (left) and receptive field sharpness (right) of individual tectal cells in response to a bar flashed at each of 5 locations across the (**B**) azimuth and (**C**) elevation axes. The maps are color-coded by the optimal stimulus position that evokes a response in each cell. Scale bar: 50 µm. (**D**, **E**) Cumulative probability distributions of receptive field sharpness of tectal cell bodies in individual animals (lighter lines) and grouped by treatment (darker lines) for the (**D**) azimuth [n = 365 cells from 6 animals for d-serine, n = 181 cells from 5 animals for control] and (E) elevation axes [n = 326 cells from 6 animals for d-serine, n = 172 cells from 5 animals for control. Kolmogorov–Smirnov test for pooled values *p < 0.05].

To investigate how d-serine-rearing may alter development of the retinotopic map, we used calcium imaging with visual stimulation to extract functional topographic maps in the optic tecta of tadpoles reared in d-serine or control media (Fig. 4A). We performed mRNA injections into one blastomere at the two-cell stage of development to generate hemilateral mosaic tadpoles expressing GCaMP6s on one side of the animal, thus labelling tectal cells but not the RGC axons innervating them from the contralateral eye. Animals were imaged at stage 48 after being reared in d-serine from stage 37, covering the developmental period during which RGC axons first arrive to innervate the tectum^50^. Two-photon calcium responses of the optic tectum were recorded while presenting receptive field mapping stimuli.

To measure the response properties of individual tectal neurons forming the retinotopic map, tadpoles were presented with a dark bar on a bright background repeatedly appearing in random order at one of 5 positions along the azimuth or elevation axes. Individual cell somata were segmented from the two-photon images and the receptive field center for each tectal cell body was estimated based on the relative strength of the responses to the stimuli in each of the 5 positions (grid map; Fig. 4B,C).

We quantified the “receptive field sharpness” of individual tectal cells to estimate their receptive field size, which has previously been shown to be enlarged by NMDAR blockade^1,59^. Receptive field sharpness is defined as the average fluorescence change to stimuli presented in the optimal stimulus positions, divided by the mean response to stimulation of the remaining non-optimal stimulus positions, for the azimuth (Fig. 4B,D) and elevation (Fig. 4C,E) axes. A higher receptive field sharpness value suggests a more compact receptive field. In the d-serine condition, there was a significant rightward shift in the cumulative probability distributions of receptive field sharpness, particularly in the elevation axis. Thus, our data suggest that d-serine rearing leads to more compact visual receptive fields for tectal neurons, consistent with arbor compaction and increased synapse density of tectal cell dendrites.

## Discussion

Our study investigated the effects of chronic administration of saturating levels of the NMDAR co-agonist d-serine on the morphological development of the retinotectal circuit. We observed that dendritic arbors of immature neurons exposed to d-serine had more compact morphology, likely due to enhanced stabilization of dendritic arbors, and had a higher synapse density. Functionally, calcium imaging revealed more compact receptive fields of tectal neurons in animals raised in d-serine. Together, these findings suggest that in the developing retinotectal circuit, maximizing activity-dependent NMDAR signalling through saturating levels of d-serine leads to the structural compression of tectal cell dendritic arbors such that they sample more extensively from a more spatially clustered set of inputs.

Our morphological observations of decreased dendritic growth under conditions of NMDAR enhancement by d-serine administration were initially surprising, given earlier reports that NMDAR blockade with APV in vivo also reduced dendritic growth over 24 h^16^ and decreased dendritic branch size and dynamics over 2 h of imaging^17^. However, our results are consistent with observations in other model systems, such as in the visual thalamus of the ferret, where dendritic arbor size and spine density were increased in response to one week of APV administration^14^. In the mammalian homologue of the tectum, the superior colliculus, NMDAR blockade leads to less refined innervation by RGC axons^13^ and enlarged receptive fields^60^. In primary cortical cultures, application of the endogenous negative NMDAR modulators kynurenate, pregnenolone sulfate, spermidine, and zinc results in dendritic arbor expansion with more distal branching^61^. Acute versus chronic drug administration may account for some discrepancies in observed effects across studies. In addition, different pharmacological agents may preferentially act on distinct cellular compartments. For example, GluN1 knockdown in postsynaptic tectal cells leads to fewer dendritic branches compared to cells in which GluN1 knockdown is targeted to their presynaptic RGC inputs^21^.

In the current study, we report that enhancing NMDAR activity with d-serine for 48 h leads to decreased arbor elaboration. This is in line with findings from the somatosensory system of mice with GluN1 knockdown, where barrelette cells receiving trigeminal whisker afferents develop longer dendrites with no orientation bias^19^. Similarly, knockout of GluN1 in cortical excitatory neurons results in exuberant dendritic arbors in spiny stellate cells that radiate without a clear bias for the barrel centers^62^. In turn, these animals show disrupted branching patterns of the whisker afferents^19,20^. Overall, loss of functional NMDARs disrupts circuit patterning throughout the somatosensory system, from the level of the brainstem^15^ to the cortex^22^.

Here, we found that enhancing NMDAR activity with d-serine for 48 h led to more compact and less dynamic dendritic arbors specifically in neurons with initially small, immature arbors. Our results showing differences between control and d-serine conditions for dendritic arbors that are initially less mature (< 500 μm initial arbor length), but not those with larger arbors (≥ 500 μm), are reminiscent of findings from previous experiments on CaMKIIα. These experiments showed that the overexpression of CaMKIIα, a key effector downstream of the NMDAR, also slows dendritic growth of immature but not of more mature neurons^63^. Thus, NMDAR activity appears to limit growth and stabilize arbors of more immature neurons, perhaps acting as an activity-dependent mediator of neuronal maturation.

To further understand how coincidence detection by NMDARs converts neuronal activity into cues for topographic refinement, one approach has been to manipulate sensory visual experience in order to induce patterned neuronal activity and study its effects in real time^9^. Specifically, stroboscopic visual stimulation can be used to physiologically drive activity in the retinotectal system^64^. Under strobe stimulation, enhanced NMDAR signaling in immature neurons exposed to d-serine for 48 h led to stabilization of dendritic branch dynamics. This decreased branch motility likely accounts for the more compact dendritic arbor morphology we observed after 48 h of d-serine exposure.

Dendritic morphology is thought to be linked to the synapses themselves. The shift from small, simple arbors to larger, more complex arbors as the neuron matures corresponds to the well-characterized shift from predominantly NMDAR-mediated glutamatergic neurotransmission typical of immature synapses to both AMPAR- and NMDAR-mediated neurotransmission at mature synapses^65,66^. Cells with shorter dendritic arbors (< 200 μm length) tend to show a lower AMPA/NMDA ratio than cells with larger arbors (> 200 μm length)^16^. Indeed, Vaughn’s synaptotropic hypothesis proposes that branch stability may be conferred by the presence of a stable synapse^67,68^. Live imaging of PSD-95-labeled synapses in zebrafish tectal neurons revealed them to be sites of dendritic stabilization from which successive branching and growth occur^69^. Here, we report increased PSD-95 punctum density following d-serine exposure. The observation that the d-serine arbors are more compact yet exhibit increased synaptic density, suggests that postsynaptic tectal neurons may experience homeostatic regulation of total synaptic input.

We chose to use d-serine rather than glycine as a more specific NMDAR co-agonist in these experiments because of the known role of glycine as an inhibitory neurotransmitter in the retinotectal system^70^. However, d-serine has also been reported to bind delta type glutamate receptors to promote synaptogenesis^57,58^. We believe that the effects of d-serine rearing in our study were mediated by NMDARs as they were rescued by the addition of the NMDAR pore blocker, MK-801 (Fig. 3F). Furthermore, our previous work has demonstrated an electrophysiological enhancement of AMPAR-mediated currents with no residual change in NMDAR currents in Xenopus tectal neurons following drug washout after 48 h of d-serine-rearing^43^. Taken together, these results indicate that the d-serine effects on tectal neurons are most likely mediated through NMDARs, and that exposure of tadpoles to saturating levels of d-serine does not lead to chronic NMDAR internalization.

These NMDAR-related changes in morphology have been tied to the development and maintenance of retinotopic maps. Chronic NMDAR blockade leads to a degradation of RGC projection convergence within the optic tectum^12^ and to desegregation of ocular dominance bands in dually innervated tecta in amphibians^7,71^. NMDAR blockade also leads to a functional enlargement of visual receptive fields in the Xenopus tectum^1,59^. Chronic pharmacological blockade of NMDARs in the visual cortex of kittens undergoing monocular deprivation prevented the loss of responsiveness to the deprived eye^72^. Additionally, chronic inhibition of GluN1 translation using antisense infusion during development prevents the maturation of orientation selectivity in the ferret primary visual cortex^73^. Overall, these lines of evidence suggest that appropriate regulation of NMDAR signaling is important for the precise targeting of axons to their dendritic partners, strengthening of appropriate synapses, and ultimately for map refinement. Our experiments, which enhanced synaptically evoked NMDAR activity, rather than relying on pharmacological blockade or knockdown, confirm and extend the conclusions of these studies on the influence of NMDAR activity on dendritic morphogenesis.

NMDAR activity also plays an important role in the fine-tuning of functional properties in the circuit. By examining tectal receptive field properties using calcium imaging, we saw that raising animals in d-serine increased receptive field sharpness of individual neurons without disrupting the organization of the coarse topographic map. This finding complements earlier work in which Xenopus optic tectal receptive fields measured electrophysiologically^59^ or by calcium imaging^1^ were enlarged as a result of chronic NMDAR blockade during the period of receptive field refinement. We therefore suggest that although NMDAR enhancement by d-serine exposure fails to grossly disrupt the overall topographic organization, largely established by molecular guidance cues^74,75^, it modulates synaptic plasticity and leads to local alterations in retinotopic response properties of tectal neurons. Interestingly, non-visual inputs from the hindbrain have been shown to target the more proximal dendrites and segregate from visual inputs by an NMDAR-dependent mechanism^76^. As we observed by Sholl analysis, that d-serine exposure leads to more compact arbors that elaborate deeper in the tectal neuropil, further investigation could reveal whether NMDAR signal enhancement by d-serine differentially affects visual versus non-visual inputs.

Finally, it will be important to investigate if endogenously released d-serine acts as a regulator of circuit plasticity under both physiological and pathological conditions. Since d-serine synthesis and release is thought to be regulated in concert by neurons^77^ and glia, particularly astrocytes^39^, this suggests a metaplasticity mechanism wherein astrocyte-regulated d-serine availability at glutamatergic synapses modulates NMDAR-mediated plasticity. Aberrant changes in NMDAR activation and d-serine synthesis and availability have been implicated in neuroinflammation and excitotoxicity in Alzheimer’s disease, amyotrophic sclerosis, ischemia, and schizophrenia^44,78^. It has been suggested that in certain pathological states d-serine regulation is abnormal, and clinical trials targeting d-serine as well as its biosynthetic and regulatory pathways are currently underway for therapeutic use^39,40^. Understanding the signaling pathways and precise mechanisms by which d-serine affects structure and function will provide important insights into experience-dependent NMDAR-mediated plasticity in both health and disease.

### Supplementary Information

Supplementary Information 1.

Supplementary Information 2.

Supplementary Information 3.

Supplementary Information 4.

## Data Availability

The custom code is available on Github for the dynamics of dendritic filopodia https://github.com/fieryzarzar/morphology_timecourse_analysis and for retinotopic mapping https://github.com/RuthazerLab/XenMap. Code for CANDLE denoising is found at https://sites.google.com/site/pierrickcoupe/softwares/denoising/multiphoton-image-filtering.
